# Minimally invasive strip craniectomy for metopic craniosynostosis using a lighted retractor

**DOI:** 10.3171/2021.1.FOCVID20123

**Published:** 2021-04-01

**Authors:** David S. Hersh, William A. Lambert, Markus J. Bookland, Jonathan E. Martin

**Affiliations:** 1 Division of Neurosurgery, Connecticut Children's, Hartford;; Departments of 2 Surgery and; 3 Pediatrics, UConn School of Medicine, Farmington; and; 4 UConn School of Medicine, Farmington, Connecticut

**Keywords:** craniosynostosis, endoscope, metopic, minimally invasive, retractor

## Abstract

Surgical options for metopic craniosynostosis include the traditional open approach or a minimally invasive approach that typically involves an endoscopy-assisted strip craniectomy. The minimally invasive approach has been associated with less blood loss and operative time, a lower transfusion rate, and a shorter length of stay. Additionally, it is more cost-effective than open reconstruction, despite the need for a postoperative cranial orthosis and multiple follow-up visits. The authors describe a variation of the minimally invasive approach using a lighted retractor to perform a strip craniectomy of the metopic suture in a 2-month-old patient with metopic craniosynostosis.

The video can be found here: https://vimeo.com/511237503.

## Transcript

In this video, we describe a minimally invasive approach using a lighted retractor to perform a strip craniectomy of the metopic suture in a patient with metopic craniosynostosis.

**0:33 History**. The patient was a 2-month-old female who was initially referred at 6 weeks of age for trigonocephaly. She was diagnosed with metopic craniosynostosis based upon her physical exam.

**0:45 Diagnosis**. In general, the diagnosis of metopic craniosynostosis is complicated by the fact that the metopic suture, unlike the other cranial sutures, undergoes physiological closure during infancy, typically between 3 and 9 months of age. As a result, imaging studies that demonstrate a closed suture are insufficient. Instead, the diagnosis depends upon a careful physical exam. Of note, isolated ridging along the metopic suture can be seen as a response to its physiological closure and does not confirm a diagnosis of craniosynostosis. Other findings must be present, including narrowing of the forehead and biparietal widening (resulting in trigonocephaly), pseudohypotelorism, and lateral orbital hypoplasia resulting in pterional constriction and pinching, particularly when viewed from above.^1^

**1:38 Indications for Surgery**. Once the diagnosis is confirmed, surgical intervention is typically recommended. The primary goal of surgery is remodeling of the cranial vault in order to optimize cosmesis, which can have profound social implications later in life. In a minority of patients with single-suture synostosis, increased intracranial pressure develops and may lead to neurodevelopmental delays. However, it remains controversial whether metopic craniosynostosis and its treatment are merely associated or actually causative of neurodevelopmental outcomes.^2–4^

**2:12 Surgical Options**. Surgical options include the traditional open approach, which involves a fronto-orbital advancement and provides immediate deformity correction, or a minimally invasive approach, which typically involves an endoscopy-assisted strip craniectomy.^3^ Having become increasingly popular since first being introduced by Jimenez and Barone in the 1990s, the minimally invasive approach has been associated with less blood loss and operative time, a lower transfusion rate, and a shorter length of stay.^5,6^ However, this approach does require the use of a postoperative cranial orthosis in order to guide remodeling of the skull as it grows.^7^ As a result, correction takes time. Despite the use of a postoperative cranial orthosis and the need for multiple follow-up visits, the minimally invasive approach has been demonstrated to be more cost-effective than open remodeling.^8^ In our practice, it is offered to all patients younger than 6 months of age, but the ideal patient is younger than 4 months, so that postoperative orthotic therapy can be initiated during the period of maximum brain growth.

**3:17 Preoperative Surface Scan**. In this case, both surgical approaches were offered to the family, and ultimately the minimally invasive option was selected. Surgery was scheduled for when the patient reached 2 months of age. A surface scan was obtained preoperatively in order to facilitate the fabrication of a cranial orthosis. The scan highlights the narrowing of the patient's forehead in comparison to her parietal region, consistent with metopic craniosynostosis.

**3:45 Positioning and Incision**. The patient was placed in the supine position on a horseshoe head holder. The neck was slightly flexed, and the eyes were protected with an adhesive dressing, though care was taken to include the nasion in the field. A curvilinear incision was planned immediately behind the hairline, just in front of the anterior fontanelle, and the hair in this region was clipped.

**4:06 Surgical Technique**. A No. 15 blade scalpel was used to incise the skin, and Bovie electrocautery was used to carry the incision down to the pericranium. A subgaleal pocket was created extending posteriorly to the anterior fontanelle and anteriorly to the nasion. A Penfield 4 was used to palpate the edges of the anterior fontanelle, which were then exposed with electrocautery, and the epidural space was dissected. Using Kerrison punches, a 1-cm strip of bone was removed, centered on the midline. A Cottonoid was placed in the epidural space, and a Leksell rongeur was used to extend the craniectomy toward the nasion. Once visualization of the distal suture became difficult, a retractor with an attached fiber-optic light source was placed in the subgaleal space, where it was used to lift up on the scalp and maintain visualization. The retractor was then secured to a table-mounted retractor arm. For this case, an endoscope was only used for videography, to document the view that is obtained with the lighted retractor. Here, you can see that with the lighted retractor lifting up on the scalp, and a malleable brain spatula protecting the dura, a clear and unimpeded view of the fused metopic suture was obtained. Bone removal then proceeded with a high-speed drill with a coarse diamond bit. The heat produced by the diamond bit minimizes bleeding by the bone edges, and irrigation is provided through an attachment to the retractor. Here, you can see the view of the single-surgeon setup, with visualization provided by the lighted retractor. A small amount of residual suture remains. Additional drilling was performed, and a remaining small bony bridge was disconnected using curettes and a pituitary. The unroofed nasal cartilage can now be visualized. It is critical that the craniectomy be taken all the way to the nasofrontal suture for complete release of the frontal bones. Here, you can see the edges of the craniectomy in relation to the lighted retractor in the subgaleal space and the malleable brain spatula in the epidural space. Upon achieving complete release, the bifrontal bones were palpably mobile.

**6:40 Closing**. The incision was then copiously irrigated with antibiotic irrigation. Meticulous hemostasis was obtained using bone wax along the bone edges, as well as topical hemostatic agents. The incision was then closed with 3-0 Vicryl galeal sutures and a running 4-0 Vicryl rapide for the skin. The drapes were removed, the scalp was cleaned, and bacitracin was applied to the incision.

**7:03 Postoperative Course**. Postoperatively, we utilized an institutional care pathway for craniosynostosis patients that had been developed by our division. The patient was brought to the PACU, where her vitals were monitored and a CBC was obtained 1 hour later. Patients who have undergone minimally invasive craniosynostosis surgery and have normal emergence from anesthesia, no seizures, no hydrocephalus, are hemodynamically stable, have an uncomplicated airway, and are found to have a postoperative hemoglobin greater than 6 are then transferred to the med/surg floor for overnight observation. Indeed, other groups have also shown that intensive care admission after the surgical repair of nonsyndromic craniosynostosis is not routinely indicated.^9^

In our patient's case, the hemoglobin level obtained 1 hour postoperatively was 8.9. She was transferred to the floor and discharged on postoperative day 1. She initiated use of a postoperative cranial orthosis on postoperative day 6 and wore the orthosis for 23 hours a day. She ultimately used two orthoses for approximately 3 months each, culminating in a total duration of 6 months. She demonstrated progressive improvement in her trigonocephaly throughout this period.

**8:16 6-Month Follow-Up**. At her 6-month follow-up, the patient was noted to have widening and normalization of her forehead.

A comparison of her preoperative images with her 6-month postoperative images confirms the improvement in forehead contour that was obtained.

**8:29 Lighted Retractor Versus Endoscope**. Notably, our surgical approach utilized a variation of the minimally invasive strip craniectomy typically described, which utilizes a neuroendoscope to assist with visualization. We, on the other hand, have adapted a right-angle retractor with attachments for a fiber-optic light source and irrigation. Attaching the retractor to the OR table facilitates a single-surgeon approach. The retractor provides the necessary visualization but is less costly and requires minimal maintenance compared to the neuroendoscope, and might be considered for centers in developing countries with limited resources.

At our center, a total of 15 patients with metopic craniosynostosis have been successfully treated in this fashion since 2015. To date, none have required revision surgery.

## Author Contributions

Primary surgeon: Hersh. Assistant surgeon: Martin. Editing and drafting the video and abstract: all authors. Critically revising the work: Hersh, Bookland, Martin. Reviewed submitted version of the work: all authors. Approved the final version of the work on behalf of all authors: Hersh.
